# Sustainable detergent-disinfectant agent based on whey mineralizate and silver nanoparticles for cleaner production in dairy industry

**DOI:** 10.1038/s41598-024-71542-9

**Published:** 2024-10-13

**Authors:** Andrey Blinov, Anastasiya Blinova, Andrey Nagdalian, Shahida Anusha Siddiqui, Alexey Gvozdenko, Alexey Golik, Zafar Rekhman, Dionis Filippov, Mohammad Ali Shariati, Ammar AL-Farga, Saleh M. Al-maaqar

**Affiliations:** 1 Department of Physics and Technology of Nanostructures and Materials, North Caucasus Federal University, Stavropol, Russia 355000; 2 Laboratory of Food and Industrial Biotechnology, North Caucasus Federal University, Stavropol, Russia 355000; 3 Independent researcher, Straubing, Germany; 4 Kazakh Research Institute of Processing and Food Industry, Semey Branch of the Institute, 238«G» Gagarin Ave., 050060 Almaty, Republic of Kazakhstan; 5 Department of Biochemistry, College of Science, University of Jeddah, 23218 Jeddah, Saudi Arabia; 6 Department of Biology, Faculty of Education, Albaydha University, Al-Baydha, Yemen

**Keywords:** Colloidal silver, Milk, Whey, Mineralization, Toxicity, Cleaner production, Biochemistry, Biotechnology, Chemical biology

## Abstract

Detergents and disinfectants for dairy industry must meet a variety of characteristics, including low toxicity, high antibacterial activity, and excellent rinsing of pollutants from working surfaces. This work presents an innovative detergent-disinfectant agent based on whey mineralizate and silver nanoparticles (Ag NPs), which allows reducing production costs and ensuring high cleanliness of treated surfaces compared to analogues. For this purpose, a method for obtaining sols of Ag NPs stabilized with didecyldimethylammonium bromide (Ag NPs-DDAB) was developed and optimized using neural network algorithms. Characterization of Ag NPs-DDAB showed particles with a radius of 4.5 nm and 20 nm, stable in the pH range from 2 to 11. An acute toxicity study of Ag NPs in mice showed LD50 = 4230 μg/kg. Based on the degree of accumulation and inhalation toxicity, Ag NPs-DDAB are classified as low-hazard chemicals. The developed detergent-disinfectant had a washability of about 90%, high antimicrobial activity (0.005 mg/mL) against *Penicillium roqueforti* and a sanitary and hygienic effect on coliforms, general contamination and pathogenic microorganisms, a low-corrosive effect and low toxicity (315 mg/mL) to *Danio rerio*. It was concluded that the use of detergent-disinfectant agent will completely eliminate the consumption of water for the equipment cleaning process and can be used to clean an electrodialysis unit’s circuits, enabling the utilization of secondary waste from membrane milk processing and promoting resource efficiency and cleaner production in the dairy industry.

## Introduction

Dairy products’ quality and safety are directly influenced by technological control throughout the entire production process, as well as by how clean and hygienic the facility is. This necessitates a particular method of cleaning and disinfecting the equipment. According to several researches, milk’s microbiological contamination with unwanted microflora rose by at least 10 times when using inadequately well-washed equipment^1–3^. In this regard, high-quality and safe dairy products will be produced on the equipment as long as the sanitary treatment of the equipment is carried out^3–5^.

The cleanliness of the milking equipment affects the bacterial contamination of milk by 80%^6^. As a result, the market has a huge selection of disinfectants. But even the best disinfectants can only complete this duty to a maximum of 80–90%^7^. After washing, the residual 10–20% of germs still represent a serious threat. As a result, only the application of logical equipment cleaning and disinfection technologies can satisfy hygienic criteria.

However, the majority of dairy businesses still employ conventional detergents and disinfectants based on sodium hydroxide, sodium carbonate, or nitric and sulfamic acids^8^. Gleeson et al.^9^ investigated the efficacy of container cleaning with sodium carbonate solutions containing 0.5–1.5% and disinfection with bleach solutions containing 150–200 mg/l of active chlorine. *E. coli* was discovered to be present on the surface of 45.7% of the objects examined prior to washing, 32% following washing, and 22.2% following disinfection. With regard to enterococci, a similar condition was seen.

Both Cremonesi et al.^10^ and McCarthy et al.^11^ report on the poor detergency of these compounds. Their findings are supported by dairy industry production practices, which include the requirement for frequent washing, which lengthens the washing process and raises the cost of chemicals and energy sources^12^. Such a detrimental aspect as the usage of water with enhanced carbonate hardness in washing procedures exacerbates the predicament of bad cleaning quality^13^.

On the surfaces of the capacitive equipment and pipelines, a substance known as “milk stone” is also forming concurrently^14^. As undesirable microorganisms develop more vigorously in “milk stones,” the product’s flavor and physicochemical characteristics suffer. The possibility of these compounds being present in milk and other dairy products is another unfavorable aspect^15^. The most common method for removing “milk stone” is disassembly, removal with scrapers, and then repeated complete washing with alkaline and acidic solutions^16^, which substantially complicates the cleaning procedure at dairy facilities.

There are currently new perspectives on cleaning techniques. For instance, Moradi and Tajik^17^ suggested a technique for using electrolyzed oxidizing water to clean the flow pipes of a dairy firm. It makes it possible to reach a suitable level of chemical and microbial purity. This approach does not, however, exhibit well detergency^18^. As active disinfectants, hypochlorite, hydrogen peroxide, and peracetic acid are also employed. Hydrogen peroxide-based detergents have the power to kill spores but can harm equipment in high quantities or at high temperatures^19^.

Similar to hydrogen peroxide, sodium hypochlorite is extremely effective against a variety of bacteria, spore cells, and viruses^20^. Antibiotics are also often employed, but they are becoming less effective because of the ability of microbes to adapt and the possibility of finding antibiotic residues in milk^21^. Among the inert and eco-friendly methods, ultraviolet radiation^22^ or ozonation^23^ can be considered, but electrophysical methods are resource- and energy-intensive, and can be used only as disinfection. All of the provided procedures, however, are only the selection and testing of already-existing, publicly-available techniques.

The various equipment types, their technical functions, and operating circumstances imply that the types of pollutants on their surfaces vary, necessitating a customized approach to sanitary treatment technology^6^. In addition to temperature and product content, other factors that affect contamination structure and removal include equipment material and technological process characteristics. These include membrane-based techniques (ultrafiltration, reverse osmosis, and dialysis) for processing milk, whey, and milk mixes. Although membrane technology increases the amount of protein used in food, it is still not widely used^24^.

It is partially caused by the difficulty in cleaning membranes properly and the dearth of powerful detergents and disinfectants for this purpose. Protein, lipid, and mineral salt deposits that accumulate on the membranes during operation lower the efficiency of ultrafiltration^25^. A nutritional media for pathogenic microorganisms is also introduced^9^. Therefore, a complete cleaning of the membranes and their disinfection are required to keep them in a sanitary and hygienic condition, to ensure the safety of the goods and the stability of the equipment.

In this work, whey mineralizate (WM) is proposed as a base of detergent for dairy equipment to address these issues. There are two reasons why WM was selected as the base. First off, WM must be disposed of in compliance with regulatory regulations as it is a waste product of the membrane processing of whey^26^. As a result, it has not yet found use in industry. Second, WM contains phosphates, potassium, sodium, and trace element chlorides, as well as organic and mineral acids in its chemical makeup. It turns the WM into a special secondary product that can be taken into consideration for various food business applications^27^. Reusing the WM will support the idea of rational raw material use and enhance the financial and resource efficiency of dairy firms, which is another factor in this choice.

It is suggested to employ silver nanoparticles (Ag NPs) as a disinfection component since detergents and disinfectants for the dairy sector must meet a variety of characteristics, including low toxicity, high antibacterial activity, and excellent rinsing of pollutants from working surfaces^28^. NPs are currently becoming more prevalent in the ingredients of different detergents and disinfectants^29,30^, and one of the most well-known antibacterial nanoscale agents is Ag NPs^31^.

The aim of this work was to develop and characterize a detergent-disinfectant agent based on WM and Ag NPs, to evaluate its functional properties, and to assess its safety using model laboratory animals: mice and Zebrafish. This work was motivated by the idea of full and rational use of milk raw materials. We were inspired to conduct this study since, as far as we are aware, there is currently no scientific data on the usage of WM as a foundation for detergents for dairy operations.

## Materials and methods

The experiments carried out relate to the production and study of the properties of nano-sized materials, as well as their use in the dairy industry. All studies were conducted in the laboratories of the North Caucasus Federal University from June 10 to October 2, 2023.

### Materials and reagents

Reagent-quality chemicals and grade A glassware were employed. Distilled water used in studies had a conductivity of less than 2 S/cm. The following chemicals were used in the work: didecyldimethylammonium bromide (Merck KGaA, Germany), silver nitrate (Petronite, Russia), sodium borohydride (Chemical Point UG, Denmark), Kolliphor HS 15, (Sigma-Aldrich Chemie GmbH, Germany), aerosil (Lenreactive, Russai), agar–agar (StavReaChem, Stavropol, Russia), *Penicillium roqueforti* strain (Danisco Deutschland GmbH, Germany). According to section “Method of WM production”, whey permeate, cheese WM, curd WM, and casein WM were created using the Stavropol Dairy Plant’s facilities in Stavropol, Russia.

### Synthesis of Ag NPs

**Fig. 1:**
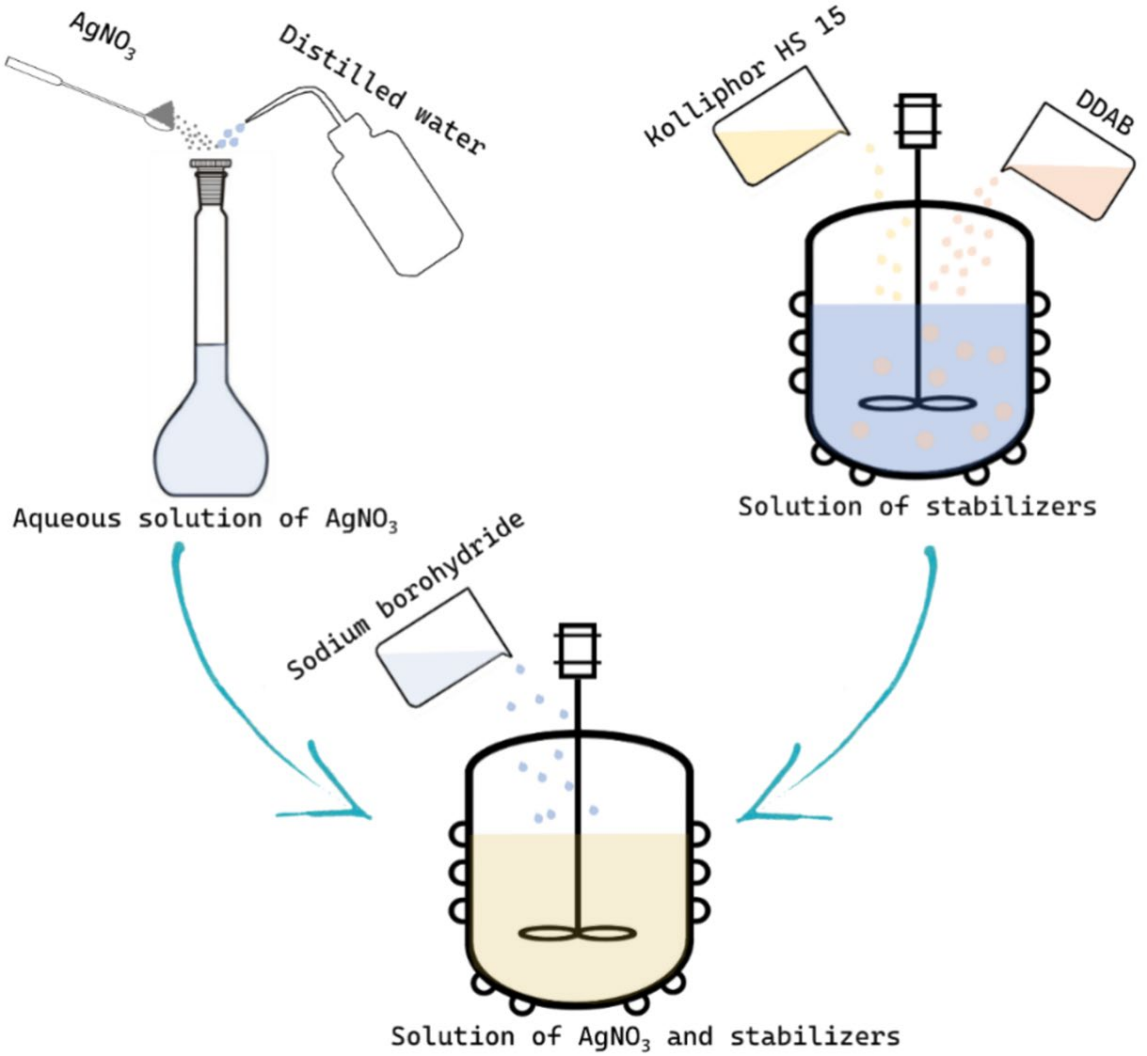
Scheme of synthesis Ag NPs-DDAB.

Figure 1 shows a scheme of synthesis of Ag NPs stabilized with didecyldimethylammonium bromide (Ag NPs-DDAB).

Ag NPs-DDAB were synthesized step by step in accordance with the pre-existing work’s approach^32^. The first step involved adding exact samples of sodium borohydride and Kolliphor HS 15 to 100 ml of a 7.8% DDAB solution while stirring continuously for 15 min to create a homogenous, transparent solution. In the subsequent step, a working solution of silver nitrate (AgNO_3_) was made using the precise weighting method. To do this, 0.385 g of AgNO_3_ was weighed separately on analytical scales M-ER 123 (Mertech, Russia) before being quantitatively transferred to a 100 ml volumetric flask and double-distilled water was added to the target. In the third stage, 10 ml of AgNO_3_ solution was gradually added to the reducing agent and stabilizer solution while vigorously stirring (1400 rpm) the mixture for an hour. Following synthesis, Ag NPs were placed in glass vials and kept at t = 5 °C for storage.

### Optimization of the synthesis process of Ag NPs

The variables (variable parameters) that have the most effects on the generation of Ag NPs in aqueous solutions of DDAB when employing sodium borohydride (NaBH_4_) as a reducing agent were identified through preliminary experiments and literature data analysis^33,34^.

The following parameters were selected as variables:

1. Mass ratio precursor: stabilizer or m(AgNO_3_): m(DDAB);
2. Concentration of DDAB or ω(DDAB), %;
3. The flow rate (υ) of the precursor into the reaction system, mL/min;
4. The molar ratio of reducing agent: precursor or n(NaBH_4_): n(AgNO_3_).

The response function (output parameter) is the biologically active fraction (BAF) of Ag NPs with hydrodynamic diameter ≤ 50 nm (Y_BAF_, %).

Preliminary studies enabled us to discover the amounts of change in variable parameters that had the greatest influence on Ag NP synthesis (Supplementary, Table S1). An orthogonal plan of 16 trials in three-fold repetition was used to explore the four components with their variation at four levels. Numerical values of the variable parameters for each experiment are also presented in Supplementary (Table S2).

**Fig. 2:**
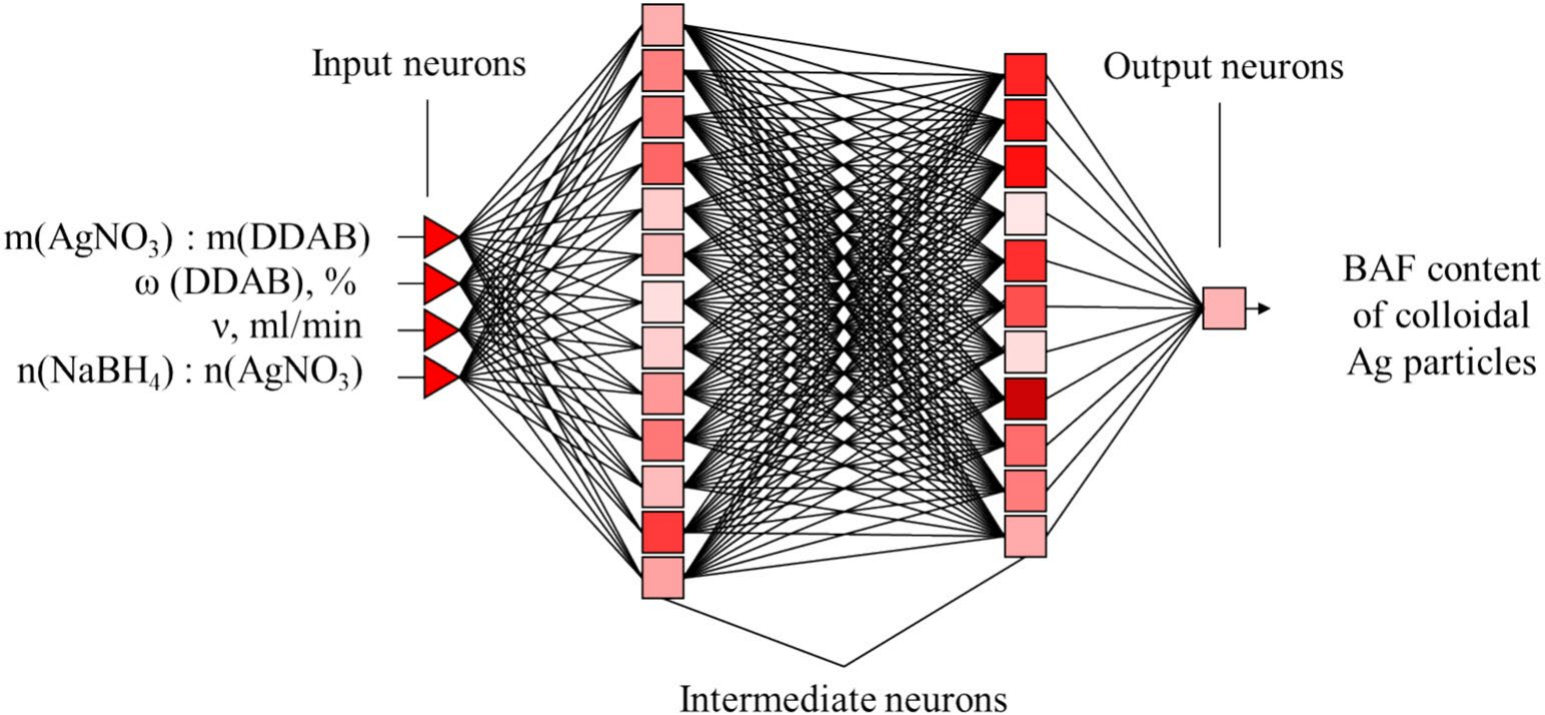
Architecture of a multilayer perceptron for determining the optimal parameters of the synthesis of Ag NPs.

The experimental data were analyzed using conventional variance, regression, and correlation techniques. Statistica 10.0 (Statsoft, Tulsa, OK, USA) software was used to automate computations for identifying gross mistakes, calculating variances, and assessing coefficient adequacy. Figure 2 shows a neural network that was developed to analyze the experimental data. It is a multilayer perceptron with four input variables and one output.

The neural network was brought in line with experimental data using training techniques. The data array was then analyzed by a neural network, and ideal parameters for the synthesis of Ag NPs were established, assuring the highest possible production of BAF.

### Investigation of Ag NPs-DDAB

#### Characterization of Ag NPs stabilized with DDAB

A UV–Visible spectroscopy (UV–Vis spectroscopy) experiment was carried out using a UNICO 2802 apparatus (United Products & Instruments, USA). Quartz cuvettes were used to hold the samples. The comparative solution was distilled water. According to Patil and Chougale, absorption spectra were collected in the wavelength range 350–610 nm^35^.

X-ray diffraction (XRD) was used to investigate the phase composition of Ag NPs samples using a PANanytical Empyrean diffractometer (PANalytical, the Netherlands). The sample analysis and preparation were carried out in accordance with Blinov et al.^36^. Before measuring, the test sample was homogeneously ground in a mortar with aerosil.

Measurement parameters:

- Copper cathode;
- Emission wavelength: 1.54 A;
- Current: 35 mA;
- Voltage: 40 kV;
- 2θ measurement range: 10–90°;
- 2θ sampling frequency: 0.01°.

Atomic force microscopy (AFM) was performed on NT-MDT Ntegra Aura atomic force microscope (NT-MDT, Russia) by a semi-contact method using an NSG01 cantilever with a cone-shaped probe (tilt angle 20°) according to Patil and Chougale^35^. For AFM, Ag NPs-DDAB were deposited by centrifugation on glass conductive base and dried at room temperature.

The determination of the average hydrodynamic radius (R_ah_) of the particles was carried out by dynamic light scattering (DLS) method on a Photocor-Complex instrument (Antek-97, Russia) with 3 repetitions. Processing of the results was carried out using the DynaLS software (Antek-97, Russia).

Measurement parameters:

• Measuring angle—90°;

• Solvent—water;

• Number of measurements per cycle—100.

Micrographs of Ag NPs-DDAB were obtained using a scanning electron microscope (SEM) MIRA3-LMH (Tescan, USA). For the investigation, the samples were dried. The sample was prepared in the following manner: a double-sided conductive carbon tape was pasted on a standard instrument table (12 mm). The conductive carbon tape was then coated with Ag NPs-DDAB powder, and a carbon coating with a thickness of around 10 nm was deposited.

The parameters of the measurement were as follows:

- Voltage 10 kV.
- Work Distance 4.9 mm.
- In-Beam SE detector.

The acid–base properties of the samples were studied by potentiometric titration on a pH meter (ionomer) Expert-001 (Econix-Expert, Russia) using a combined electrode LE409 (Mettler Toledo, Russia).

The electrokinetic potential (ζ-potential) was determined by acoustic spectroscopy on DT 1202 spectrometer (Dispersion Technology, USA).

#### Study of Ag NPs-DDAB toxicity

Study of acute toxicity of Ag NPs-DDAB was carried out on 60 clinically healthy white laboratory mice, which were in the same conditions of maintenance and feeding. Six groups of laboratory mice with 10 individuals each were formed. The first group served as a control, laboratory mice of the second, third, fourth, fifth and sixth groups were orally injected with Ag NPs-DDAB in increasing doses from 4000 to 5000 µg/kg in compliance with the rules of asepsis and antiseptics. The control animals were injected with an appropriate volume of distilled water. During the experiment, the following toxicity parameters were determined: the minimal lethal dose (MLD), the median lethal dose (LD_50_), the doses of the effect of LD_16_ and LD_84_ and the error index of LD_50_ (SLD_50_).

To carry out an accelerated determination of the cumulative effect of Ag NPs-DDAB, 2 groups of white laboratory mice (n = 10) with a live weight of 20–22 g were formed. Preparation of Ag NPs-DDAB was orally administered to laboratory animals of the first group, once a day. The dosage of preparation was calculated based on the results of acute toxicity experiment: 0.1LD_50_ on the first 4 days, 0.2 LD_50_ on the 5–9th days, 0.4 LD_50_ on the 9–14th days, and 0.5LD_50_ from the 15th day of the experiment. The second group animals received distilled water in equal volume with Ag NPs-DDAB preparation administrated to mice from the first group. The experiment was carried out for 21 days with registration of changes in appearance and behavior of mice, indicating intoxication.

To determine the inhalation toxicity of Ag NPs-DDAB, 2 groups of white laboratory mice (n = 10) with a live weight of 20–22 g were formed. Before the experiment, laboratory mice were weighed and placed in containers with a closed lid, which were laboratory desiccators. The first group desiccators contained Ag NPs-DDAB preparation, and the second group was a control. During the experiment, behavioral features of laboratory animals were analyzed and a live weight changes were recorded.

#### Microbiological tests

In order to determine the MLD of Ag NPs-DDAB, microbiological tests were carried out using the method of serial dilutions on a liquid nutrient medium. *Penicillium roqueforti* was used as a mold culture.

### Method of WM production

**Fig. 3:**
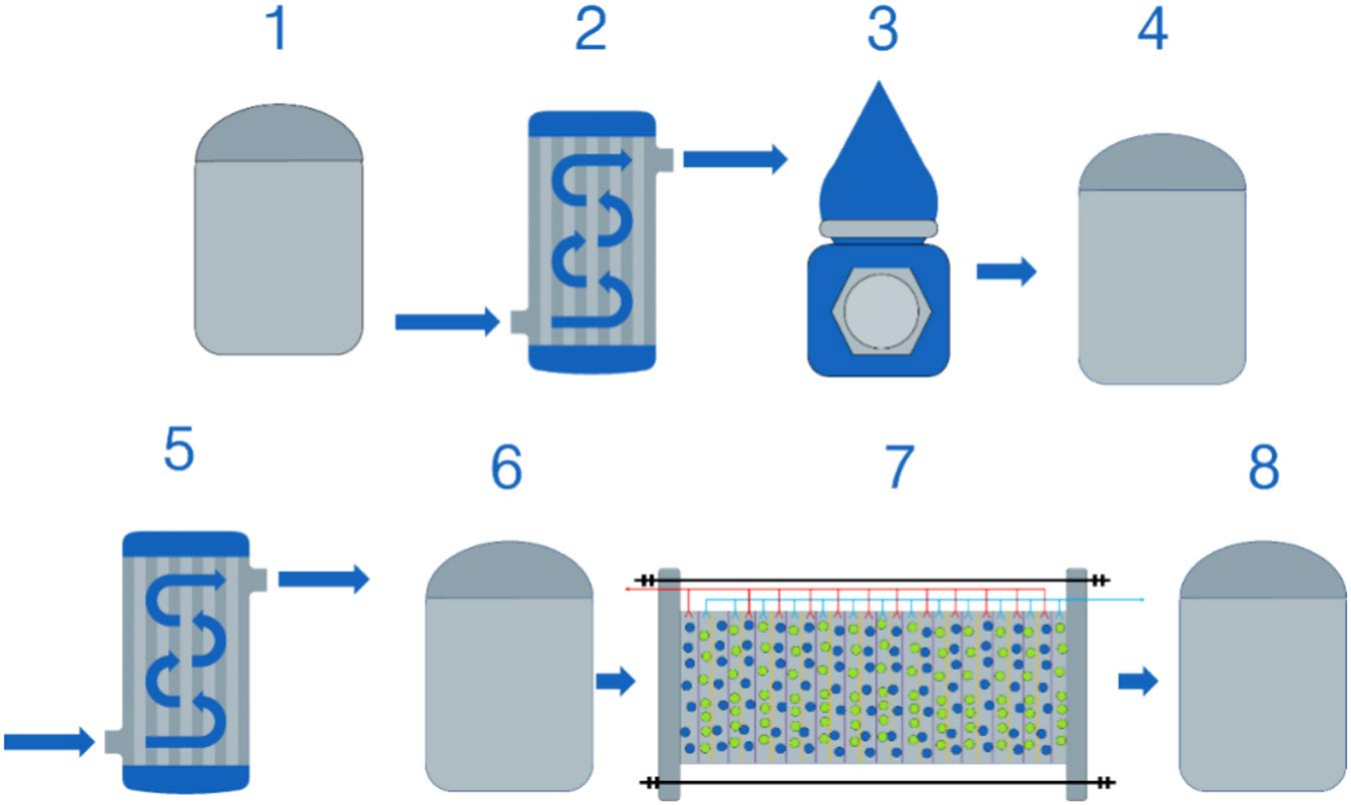
Technological scheme of WM production. 1,4,6,8—tank; 2 and 5—pasteurization and cooling unit POU-3000 (E8, Russia); 3—cream separator Zh-5-OSCP-3 (UralPromLuch, Russia); 7—electrodialysis unit ED mini (MEGA, Czech Republic).

Figure 3 shows a scheme of WM production.

Furthermore, in the pasteurization and chilling unit (5), whey was pasteurized at 74 ± 2 °C for 15 s before being cooled to 8 ± 2 °C. Cooled whey was kept in tank (6) for periodic processing in the electrodialysis unit (7). At 14 ± 2 °C, whey was demineralized to the appropriate amount before being cooled to 6 ± 2 °C. Demineralization was performed using RALEXAMH-PES anionite membranes and RALEXCMH-PES cationite membranes using an ED micro electrodialysis device (MEGA, Czech Republic). At the end, WM was collected in tank (8) for use in the experiment.

Whey permeate, cheese WM, curd WM, and casein WM were made for the experiment using this approach on the basis of Stavropol Dairy Plant. The relationship of physicochemical parameters (electrical conductivity and pH) on degree of mineralization of cheese WM, curd WM, and casein WM on degree of mineralization was researched to establish the appropriate foundation of the detergent and disinfection agent.

### Preparation and study of detergent-disinfectant agent based on WM and Ag NPs-DDAB

#### Preparation of detergent-disinfectant agent

To analyze the WM as a solvent for synthesized NPs we measured R_ah_ of Ag NPs-DDAB, dissolved in WM, distilled water, whey permeates, tap water and 1 M and 5 M NaCl solutions. Measurement of the average hydrodynamic radius of NPs was carried out by DLS method with Photocor-Complex instrument (Antek-97, Russia).

As solvents for the creation of a detergent-disinfectant agent, distilled water, tap water, and curd WM were utilized. The concentration of Ag NPs-DDAB was 0.1%. In prepared solutions, the active pH acidity, contact wetting angle, and surface tension were all determined.

#### Antimicrobial activity of detergent-disinfectant agent

The disc diffusion technique was used to assess the sensitivity of *Penicillium roqueforti* to Ag NPs-DDAB solutions. The experimental solvents were cheese WM, curd WM, and casein WM, while tap water served as the control solvent. The spore suspension was sown on the nutritional medium’s surface (agar–agar). The prepared culture samples were then covered with 10 mm paper disks, and an equivalent volume of Ag NPs-DDAB was administered at the following concentrations: 0.5 mg/mL, 0.05 mg/mL, 0.005 mg/mL, and 0.0005 mg/mL. The samples were then incubated at 25 °C in thermostat TSO-1/80 SPU (LabTech, Russia). The suppression of *Penicillium roqueforti* was assessed after two days of incubation by measuring the diameters of the zones (in millimeters) surrounding the discs using a caliper.

#### Sanitary treatment properties of detergent-disinfectant agent

To assess the quality of hygienic treatment of the working surface with Ag NPs-DDAB, a model experiment was carried out. Ag NPs-DDAB (0.5 mg/mL) working solutions in cheese WM and curd WM, as well as a commercial alkaline detergent used by Stavropol Dairy Plant (Stavropol, Russia), were the topics of the investigation. At a working solution temperature of 50–55 °C, the model experiment was carried out utilizing circulation washing. Glass, stainless steel, and aluminum plates contaminated with 20% fat sour cream served as work surfaces. During the experiment, outwashes from the working surface were collected. As microbiological indicators, coliforms, total bacterial count (TBC), and pathogenic microorganisms such as salmonellas were discovered.

#### Corrosive properties of detergent-disinfectant agent

The corrosive characteristics of Ag NPs-DDAB dissolved in cheese WM, curd WM, and casein WM were investigated using aluminum and stainless-steel plates. As a comparative medium, tap and distilled water were utilized. During the experiment, 25 × 25 mm metal plates were submerged in the test liquid for 100 h at 20, 35, and 50 °C. The corrosion rate was calculated using the weight technique, which was based on the difference in the mass of samples before and after the experiment, which was assigned to the surface area exposure time. The corrosion rate (mm/year) was calculated using the following formula (1):

1 $$C = \frac{8760 \cdot L}{{\rho \cdot 1000}}$$

where: C is the corrosion rate, mm/year; L is the loss of metal weight, g/(m^2^·h); 8760 are calculated hours per year (324 × 24), h; $\rho$ is a metal density, kg/m^3^.

#### Toxicity of detergent-disinfectant agent

To study the toxicity of the developed detergent-disinfectant agent in relation to hydrobionts, 140 *Danio rerio* (shortfin phenotype of Zebrafish) were used as test objects and kept in 5-L aquariums (n = 5 fish per aquarium) for two weeks before the start of the experiment. The water was restored and buffered with Mydor Target 7.0 buffer to pH = 7.0. Constant filtration, temperature control (27 ± 1°C), lighting (14/10 h, cycle start at 07:00 am) and timely feeding (Oscar Gold granulated diet) were provided in the aquariums. The conditions for raising and keeping animals corresponded to the standards established by ASAB/ABS^37^. The created detergent-disinfectant agent and commercial alkaline detergent were used as the analysis samples, while drinking tap water served as the control. Tests in vivo were conducted for 96 h (without feeding). Every day, the dead fish were removed and the number of alive fish was counted. The analysis followed Han and Jung’s recommendations^38^.

### Statistical analysis

All parameters obtained were submitted to the Student’s *T*-test (*p* < 0.05) analysis through the statistical package STATISTICA for Windows (Statsoft, Tulsa, USA). Microsoft Excel 2010 and Origin software were used for histograms and graphs creation based on the data obtained.

## Results and discussion

### Synthesis and characterization of Ag NPs-DDAB

At the first stage of the experiment we established the optimal conditions for obtaining aggregative stable Ag NPs with sizes ≤ 50 nm. They are:

- m(AgNO_3_) : m(DDAB) = 2.6
- ω(DDAB) = 0.12%
- υ (AgNO_3_) = 4 ml/min
- n(NaBH_4_): n(AgNO_3_) = 2.5.

Following data analysis, the following regression dependency was constructed, which demonstrates the effect of factors and interfactory interactions that substantially affect the synthesis of Ag NPs stabilized with DDAB:

$$\begin{aligned} Y = & f\left( {a, b,c,d} \right) = - 33.35 - 143.20a - 107.24b - 0.17c + 229.35d \\ & + \,305.09ab + 1.90ac + 7.64ad + 36.70bc - 355.84bd + 0.35cd \\ & + \,31.97a^{2} - 67.46b^{2} - 0.14c^{2} - 66.15d^{2} \\ \end{aligned}$$

where a, b, c, d are variable parameters of the synthesis process.

Fischer’s criterion was used to evaluate the appropriateness of the generated equation. The probability was 0.95 at a significance level of 0.05. The results were extremely reproducible, and the estimated values were totally adequate, according to the laboratory-based approval of optimum parameters. Through the mathematical analysis of experimental data, connections between the BAF content in the samples and various synthesis factors were found.

**Fig. 4:**
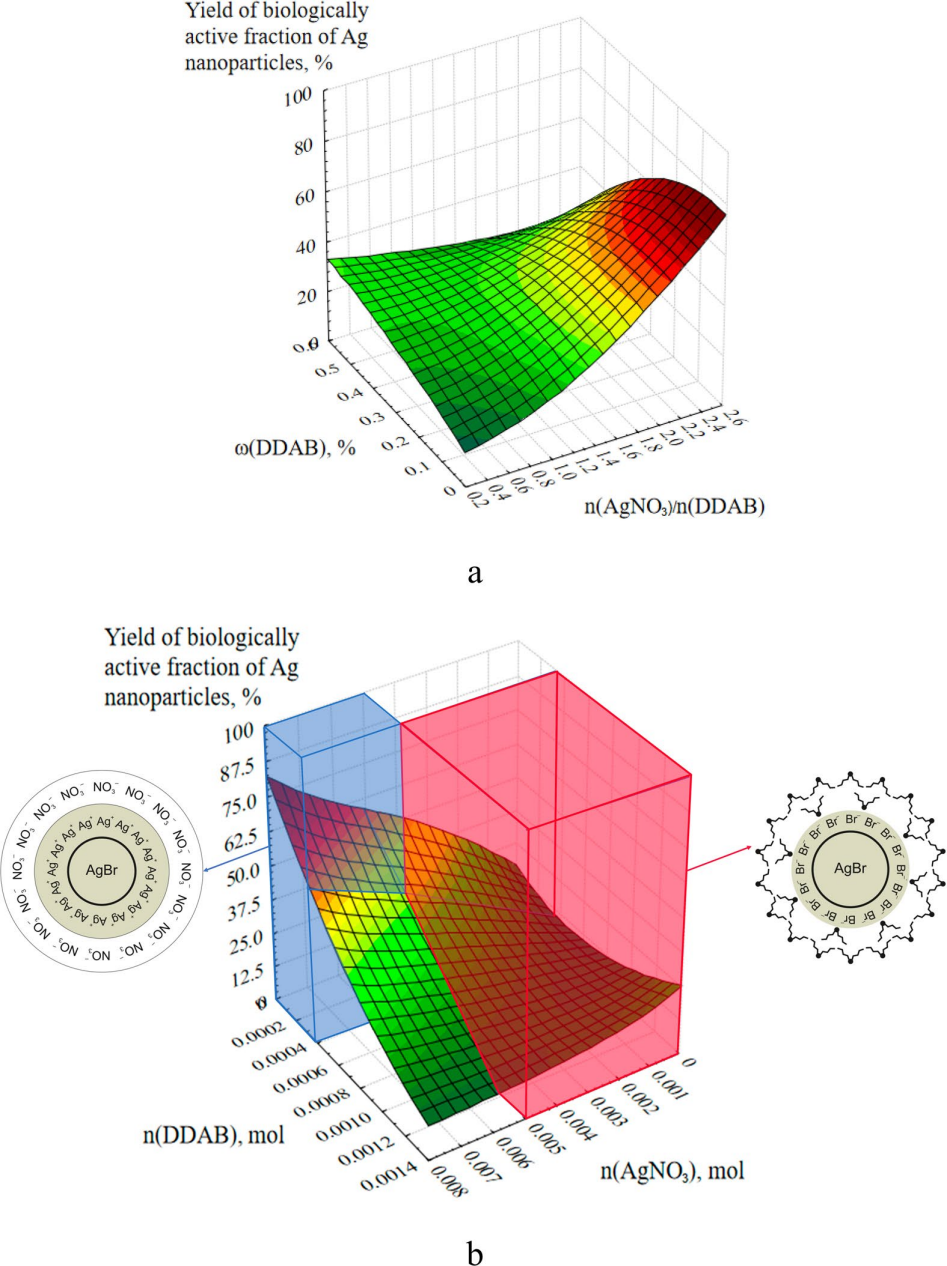
Response surfaces of the output parameter Y_BAF_ depending on the mass fraction and concentration of DDAB and AgNO_3_: dependence of Y_BAF_ on the molar amounts of DDAB and AgNO_3_ (**a**); dependence of Y_BAF_ on the concentration of ω(DDAB) and the mass ratio m(AgNO_3_) : m(DDAB) (**b**).

The response surfaces of the output parameter were built based on the mass fraction and concentration of DDAB and AgNO_3_ to provide a more visual interpretation of these data (Fig. 4). In accordance with the molar amounts of the constituents present in the reaction mixture, Fig. 4a depicts the dependence of the yield of the BAF of Ag NPs (Y_BAF_) on the mass fraction of DDAB and the mass ratio of the precursors AgNO_3_ and DDAB. Figure 4b depicts the output parameter Y_BAF_ response surface as a function of the concentration ω(DDAB) and the mass ratio m(AgNO_3_): m(DDAB).

To explain the response surface shown in Fig. 4b, it is necessary to consider the dependence of the structure of AgBr sol particles formed by mixing AgNO_3_ and DDAB on the molar ratios of reagents. Thus, in the region of high concentrations of AgNO_3_ (from 0.005 to 0.008 mol) and small concentrations of DDAB (from 0.00001921 to 0.0004 mol), a positively charged AgBr sol is formed, the structure of the micelle of which is shown in Fig. 4b. Because the potential determining layer in the forming AgBr micelles is formed due to Ag^+^ ions, which are present in excess in the solution and have the greatest affinity for the particle.

Accordingly, in this region, a large number of Ag^+^ ions are present in the solution, capable of directly participating in the formation of a low-dimensional BAF of Ag NPs, and an insignificant number of AgBr NPs that do not particularly affect this process. With a further increase of DDAB and a decrease of AgNO_3_, there will be an increase in the number of AgBr NPs and a decrease in free Ag^+^ ions. Thereafter the content of the BAF will decrease, which we observe in Fig. 4b. When the stoichiometric ratio of DDAB and AgNO_3_ introduced into the reaction mixture is reached, NPs with practically no charge will be formed. The absence of electrostatic stabilization leads to rapid aggregation of such particles^39^. As can be seen from Fig. 4b, in this concentration range, the content of the BAF is close to zero. With a further increase in the predominance of Br^−^ ions in the solution, a negatively charged AgBr sol is formed, the structure of the micelle of which is also shown in Fig. 4b.

A slight increase in the content of the low-dimensional fraction of Ag NPs may be due to the appearance of Ag^+^ ions in the solution due to dissociation:

$${\text{AgBr}} \leftrightarrow {\text{Ag}}^{ + } + {\text{Br}}^{-} .$$

The decrease in the yield of BAF of Ag NPs with an increase in DDAB content in the system may also be due to the tendency of DDAB to form its own micelles of various shapes and structures, which is energetically beneficial to the system seeking to reduce its internal energy^40^.

**Fig. 5:**
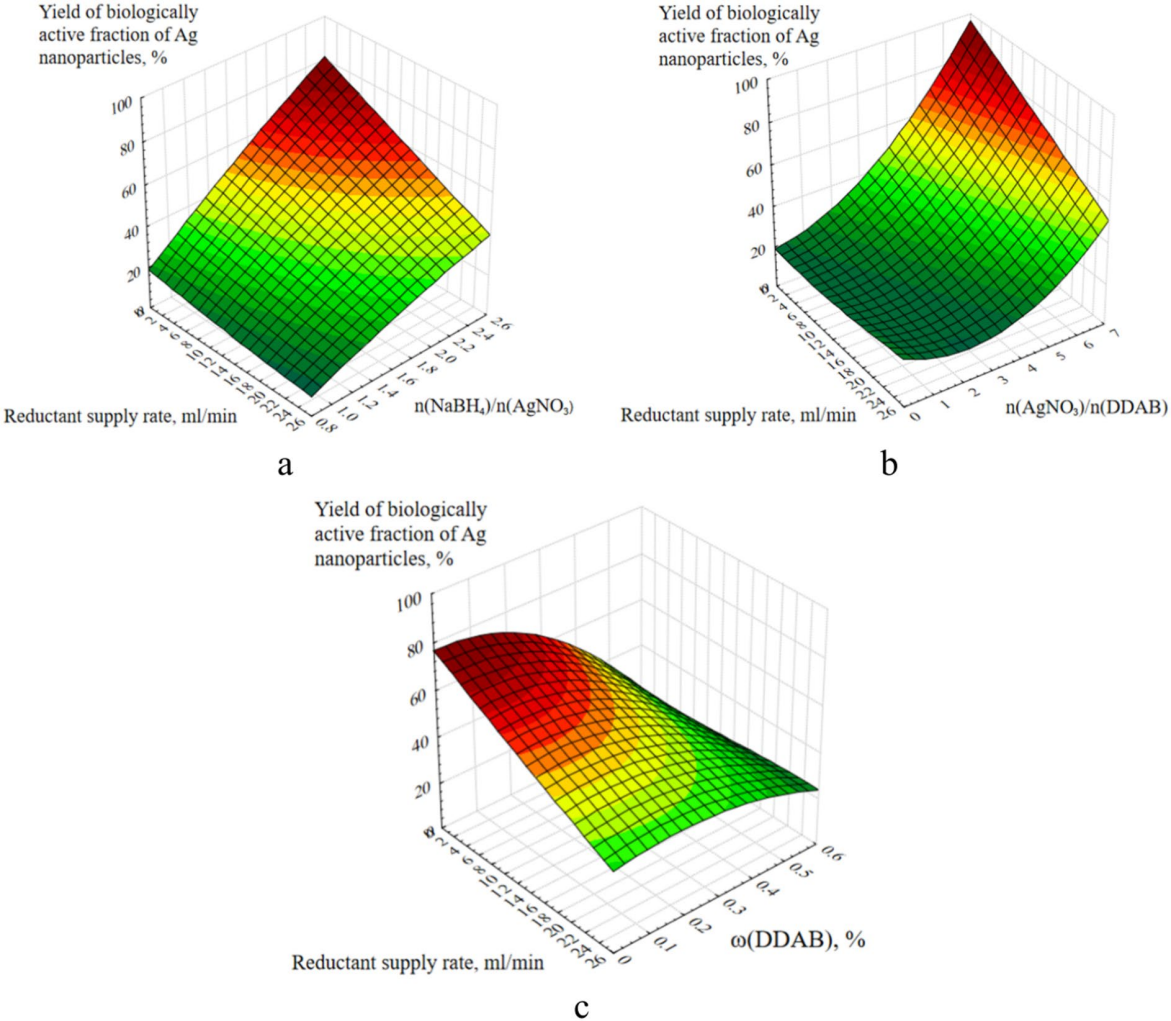
Response surfaces of Y_BAF_ depending on the flow rate of NaBH_4_ and the ratio: n(NaBH_4_): n(AgNO_3_) (**a**); n(AgNO_3_): n(DDAB) (**b**) and the mass fraction ω(DDAB) (**c**).

As a result of mathematical processing of experimental data, we obtained the response surfaces of Y_BAF_ depending on the flow rate of NaBH_4_ and the ratio: n(NaBH_4_) : n(AgNO_3_) (Fig. 5a); n(AgNO_3_) : n(DDAB) (Fig. 5b) and ω(DDAB) (Fig. 5).

A substantial molar excess of NaBH_4_ over AgNO_3_ and AgNO_3_ over the DDAB, as well as flow rates of NaBH_4_ of 8 ml/min, are required to generate the maximum content of BAF of Ag NPs, as shown in Fig. 5. These findings are supported by the fact that many Ag NPs nuclei will form in the reaction mass when mixing solutions with high concentrations of NaBH_4_ and AgNO_3_, and the low flow rate of NaBH_4_ enables the newly created NPs to be completely stabilized^33^.

**Fig. 6:**
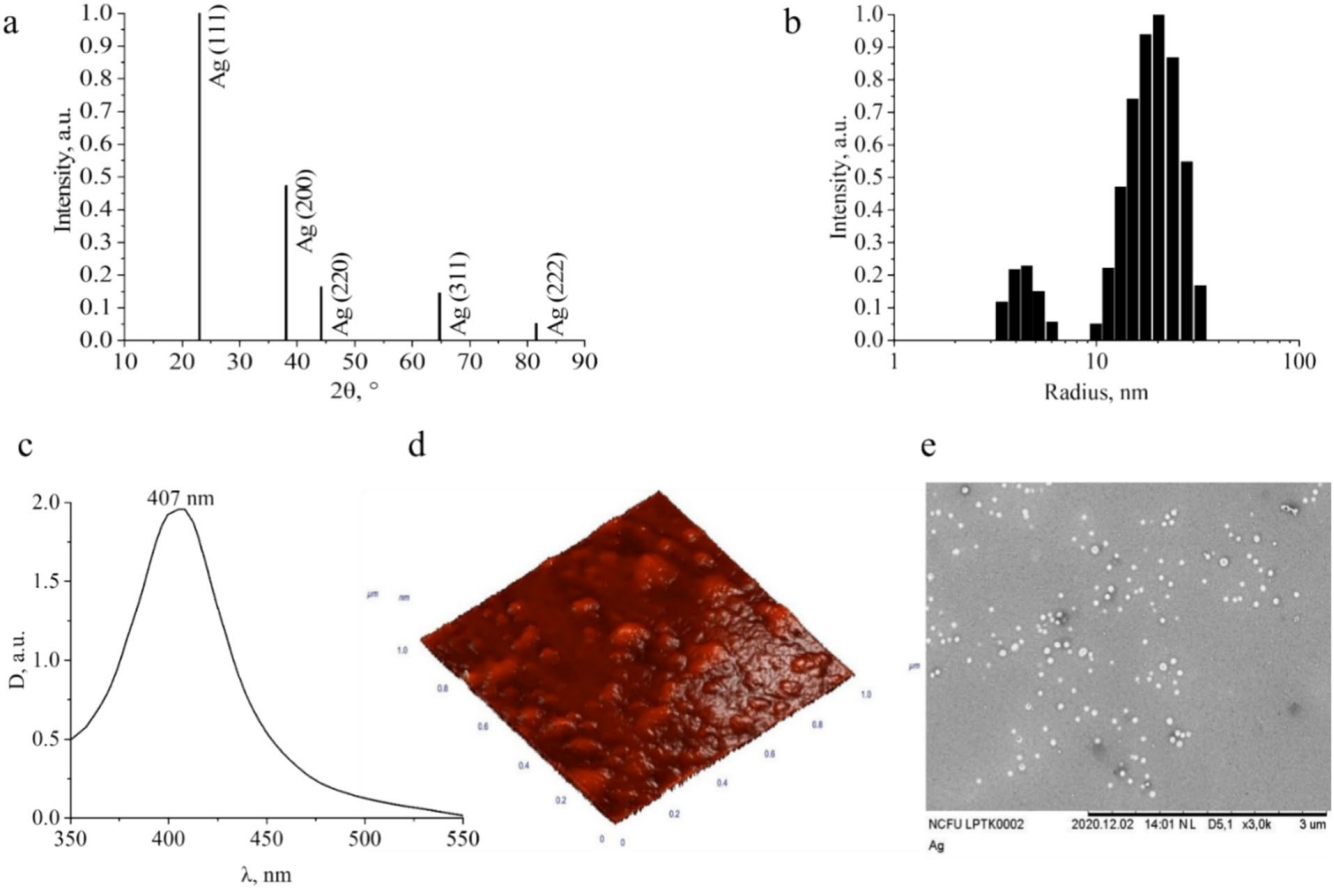
Characterization of synthesized Ag NPs: X-ray diffractogram (**a**), histograms of hydrodynamic radius distribution (**b**), absorption spectrum (**c**), AFM-scan (**d**), SEM-micrograph (**e**).

Ag NPs-DDAB were created and researched in accordance with the results found (Fig. 6). Interpretation of XRD (Fig. 6a) showed that Ag NPs have a face-centered cubic crystal lattice (space group Fm-3 m), which corresponds to results of Barke et al.^41^. During photon correlation spectroscopy (Fig. 6b) two fractions with R_ah_ of 4.5 and 20 nm. Ag NPs have a monodisperse size distribution, which was also declared by Hu et al.^42^. Acoustic spectroscopy also showed that Ag NPs have a narrow lognormal size distribution with an average diameter of about 35 nm. The small size and small polydispersity of Ag NPs is also confirmed by absorption spectrum in the UV–vis. spectral regions. The absorption spectrum has a band with an absorption maximum at 407 nm (Fig. 6c), the presence of which is due to the surface plasmon resonance of sufficiently small Ag NPs^43^. The analysis of AFM-scan (Fig. 6d) and SEM-micrograph (Fig. 6e) showed that the particles have a spherical shape with a diameter of about 40–50 nm, which confirms our results and roughly corresponds to the results of other researchers^44,45^.

Thus, a highly dispersed Ag NPs-DDAB system with determined characteristics and potential biological activity was obtained. Despite this, it is well known that the industrial use of Ag NPs is most often limited by the short-term stability. Usually, during long-term storage of Ag NPs, oxidation, ionization, agglomeration and aggregation can occur, which greatly affects their physicochemical properties^46^. Therefore, before proceeding to the next stage of research, we needed to assess how highly stabilizing agent is DDAB.

**Fig. 7:**
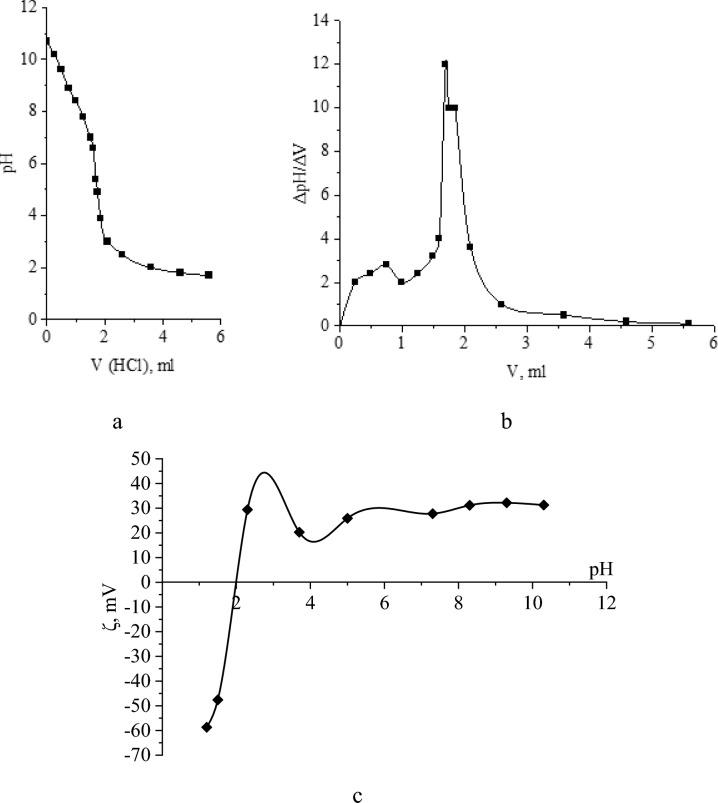
Acid–base properties and electrokinetic potential of Ag NPs-DDAB: integral titration curve (**a**); differential titration curve (**b**); dependence of the ζ-potential on pH (ζ = f(pH)).

The obtained samples were stored in a dark closed container at room temperature. During storage, histograms of Ag NPs size distribution were taken once a week. It was found that the content of BAF of Ag NPs in the samples changed slightly. Thus, optimized Ag NPs-DDAB have a high aggregative stability. To confirm it we carried out study of acid–base properties and electrokinetic potential (Fig. 7). The titration curves are shown in Fig. 7a,b.

Analysis of titration curves of Fig. 7a,b showed that Ag NPs-DDAB have a highly alkaline reaction of the medium (pH ≈ 11). The nature of the titration curves corresponds to the titration of a strong base with a strong acid with a well-defined equivalence point on both the integral and differential titration curves. The main properties of Ag NPs are due to the presence of a quaternary ammonium compound in it—DDAB^47^. The experimentally found amount of base in the synthesized Ag NPs-DDAB corresponds to the theoretically calculated amount (0.065 g/mL). Thus, Ag NPs-DDAB can be considered for use as part of an alkaline detergent under the condition of proven stability.

In order to study the stability of Ag NPs-DDAB in a wide pH range, the dependence of ζ-potential on pH was measured (Fig. 7c). The analysis of the dependence ζ = f(pH) showed that ζ-potential in the pH range from 2 to 11 practically remains unchanged and is on the order of + 25 mV. With further acidification, a change in ζ-potential from + 30 to − 60 mV was observed, that means that the surface of Ag NPs was recharged without visible signs of coagulation and without loss of stability. The data obtained indicate the high stability of Ag NPs-DDAB in the studied pH range. The synthesized samples turned out to be ultra-stable^48^.

### Safety assessment of Ag NPs-DDAB

The ultra-stable colloidal system Ag NPs-DDAB was the result of the initial research stage. However, it is vital to demonstrate the synthesized complex’s safety before utilizing it in detergents and disinfectants. Due to Ag NPs’ reported toxicity, this is especially significant^49^. The production technique, stabilizer, and stability of the colloidal solution all affect Ag NPs’ toxicity^50^. Ag NPs stabilized with citrates^51^, polyvinylpyrrolidone^52^ and even green synthesized Ag NPs^53^ have all been shown to be hazardous in several directions. The safety of Ag NPs-DDAB was therefore evaluated in the following phase of the investigation. Studies on acute toxicity, cumulative impact, and inhalation toxicity were completed for this reason.

Table 1 displays the outcomes of the mouse study on acute toxicity. In Supplementary (Table S3), a thorough explanation of the experiment’s design and data processing is provided.

**Table 1:** Acute toxicity of Ag NPs-DDAB with a single oral administration.

Administration	Toxicity parameters					SLD_50_
	MLD	LD_16_	LD_50_	LD_84_	LD_100_	
*per os*, µg/kg	3600	3857	4230	4543	4800	± 11.43

where: MLD—the minimal lethal dose, LD_16_, LD_50_, LD_84_, LD_100_—values of the lethal doses, SLD_50_—the error index of LD_50_.

LD50 was attained with the addition of 4230 g/kg of Ag NPs-DDAB, while LD100 was already reached at 4800 g/kg, per the results obtained. Because of this, the average lethal toxic dose of manufactured Ag NPs-DDAB is much higher (by 27 to 44 times) than the lethal toxic doses of Ag NPs stabilized with citrates or polyvinylpyrrolidone, which were synthesized and studied by Yaqub et al.^50^, Mao et al.^51^, Recordati et al.^52^ and Han et al^54^. Since other morphological and physico-chemical properties of the Ag NPs we produced roughly matched those of the Ag NPs synthesized by the cited authors, it is most likely caused by the ultrastabilization of Ag NPs-DDAB. According to the GHS classification, Ag NPs-DDAB might be categorized as a moderately harmful drug^55^.

Additionally, a test was done to determine the cumulative effect more quickly. Both the experimental and control groups exhibited excessive activity throughout the experiment. Both groups of mice displayed calmer behavior by the time the experiment was through. Both the death of experimental animals and any apparent harmful signs were absent. Ag NPs-DDAB can therefore be classified as moderately hazardous compounds under the GHS classification^55^ and the third (moderate) group under the Russian State Standard^56^ based on the results obtained.

Within 14 days, a new mouse observation station was constructed. No changes in the behavior of the animals were noticed during the entire monitoring period, and no deaths were noted. Mice were weighed on the first and end days of the experiment. The experiment’s findings revealed that neither group’s body weight changed significantly (Supplementary, Table S4). Based on the analysis of the data, it can be deduced that Ag NPs-DDAB does not exhibit inhalation toxicity within the studied concentration range and, based on the inhalation effect, is classified as a low-hazard substance under the GHS^56^, and the 4th hazard class under the Russian State Standard^55^.

Therefore, there should be no adverse effects on the respiratory systems of those in charge of cleaning and disinfecting equipment at dairy operations when using a detergent and disinfection agent based on Ag NPs-DDAB.

### Physico-chemical properties of WM

The best basis for the detergent and disinfecting agent was determined at the following stage, before the detergent and disinfectant agent was prepared and tested. Table 2 displays the findings of the investigation into the physico-chemical characteristics of WM.

**Table 2:** Physico-chemical parameters of WM.

WM diluate	Degree of demineralization, %	Whey		Salt concentrate	
		SEC, mS/cm	pH	SEC, mS/cm	pH
Casein WM	0	9.94	5.2	0.51	8.1
	30	6.96	5.43	1.9	6.8
	50	4.97	5.81	3.47	5.9
	70	2.98	6.34	5.67	5.44
	85	1.49	6.74	7.44	5.6
Cheese WM	0	5.32	7.84	0.56	8.32
	30	3.73	7.65	1.5	7.05
	50	2.66	7.47	2.34	7.14
	70	1.6	6.95	3.64	7.45
	85	0.8	6.56	4.69	7.74
Curd WM	0	8	6.12	0.72	8.6
	30	5.6	6.37	3	7.53
	50	4	6.53	4.27	7.02
	70	2.5	6.65	5.72	6.77
	85	–	–	–	–

Where SEC is specific electrical conductivity.

Additionally, we investigated R_ah_ of Ag NPs-DDAB, dissolved in WM, distilled water, 1 M and 5 M NaCl solutions, whey permeates, and tap water (Supplementary, Fig. S1) to evaluate the WM as a solvent for the proposed formulation. According to studies, WM can be employed as a solvent and has little impact on the Rah of Ag NPs-DDAB. On the other hand, because to the relatively high ion concentration in tap water and 5 M NaCl solution, Ag NPs-DDAB were destabilized and Rah increased to 400 and 870 nm, respectively.

The following variables were measured: pH, contact wetting angle (θ), and surface tension (σ) during the investigation of the surfactant and detergent characteristics of Ag NPs-DDAB in diverse mediums. Table 3 presents the findings.

**Table 3:** Physico-chemical characteristics of the studied solutions.

Solution	pH	Contact wetting angle, θ, °	Surface tension, σ, mN/m
Distilled water	6.67	105	71.96
0.1% Ag NPs-DDAB in distilled water	9.05	70	64.15
Tap water	7.33	97	69.11
0.1% Ag NPs-DDAB in tap water	8.78	45	40.27
Curd WM	5.05	91	70.96
0.1% Ag NPs-DDAB in curd WM	5.84	35	50.77
Cheese WM	5.92	95	69.56
0.1% Ag NPs-DDAB in cheese WM	6.53	41	48.82
Casein WM	4.01	90	68.17
0.1% Ag NPs-DDAB in casein WM	5.09	39	44.22

According to an analysis of the data in Table 3, tap water has a slightly alkaline pH, cheese WM and curd WM have an acidic pH, and distilled water has a slightly alkaline pH. A small pH change was seen when Ag NPs-DDAB were dissolved in the investigated solvents, indicating their ability to act as a buffer because of their ionic composition. The pH has drastically shifted to the alkaline side in distilled water.

The information on variations in and are of particular interest for evaluating the physico-chemical characteristics of solutions. For instance, all solvents originally had high values of θ≈100° and σ≈70 mN/m, indicating their “poor” washing capabilities^57^. Ag NPs-DDAB significantly reduced (from 60 to 20°) and (from 10 to 30 mN/m), which unquestionably should boost the interaction of the detergent with the contaminated surface and, as a result, increase washing ability.

## Research of the developed detergent-disinfectant agent

**Fig. 8:**
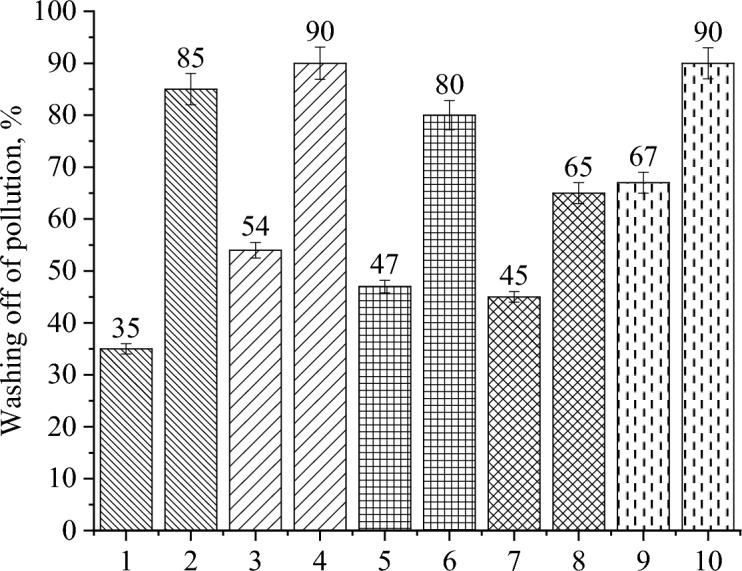
Rinsability of contamination by different solutions: 1—distilled water, 2—0.1% Ag NPs-DDAB in distilled water, 3—tap water, 4—0.1% Ag NPs-DDAB in tap water, 5—curd WM, 6—0.1% Ag NPs-DDAB in curd WM, 7—cheese WM, 8—0.1% Ag NPs-DDAB in cheese WM, 9—casein WM, 10—0.1% Ag NPs-DDAB in casein WM.

The statistics mentioned above only hinted at the detergent-disinfectant agent’s washing potential. Therefore, model research on the capacity to rinse contaminants off of glass surfaces was conducted. Figure 8 displays the research’s findings.

The examination of the data in Fig. 8 supported the preliminary findings about the enhancement of the detergent characteristics of solutions with the addition of Ag NPs-DDAB that had been made. Therefore, the rinsability of contaminants in distilled and tap water as well as in casein WM reached nearly≈ 90%, indicating a strong washing ability of these solutions^58^.

Although the data in Table 3 demonstrate that the change in contact wetting angle and surface tension increase with the addition of Ag NPs-DDAB, the mineralizate of curd WM and cheese WM showed lower rinsability. It’s probable that the curd WM and cheese WM’s moderately acidic medium interaction is responsible for the contamination’s low rinsability. As is common knowledge, detergents with an extremely alkaline or extremely acidic medium reaction are used in the milk industry to wash away fatty impurities by hydrolyzing fat, which is characterized by fatty impurities in dairy production^59^.

**Fig. 9:**
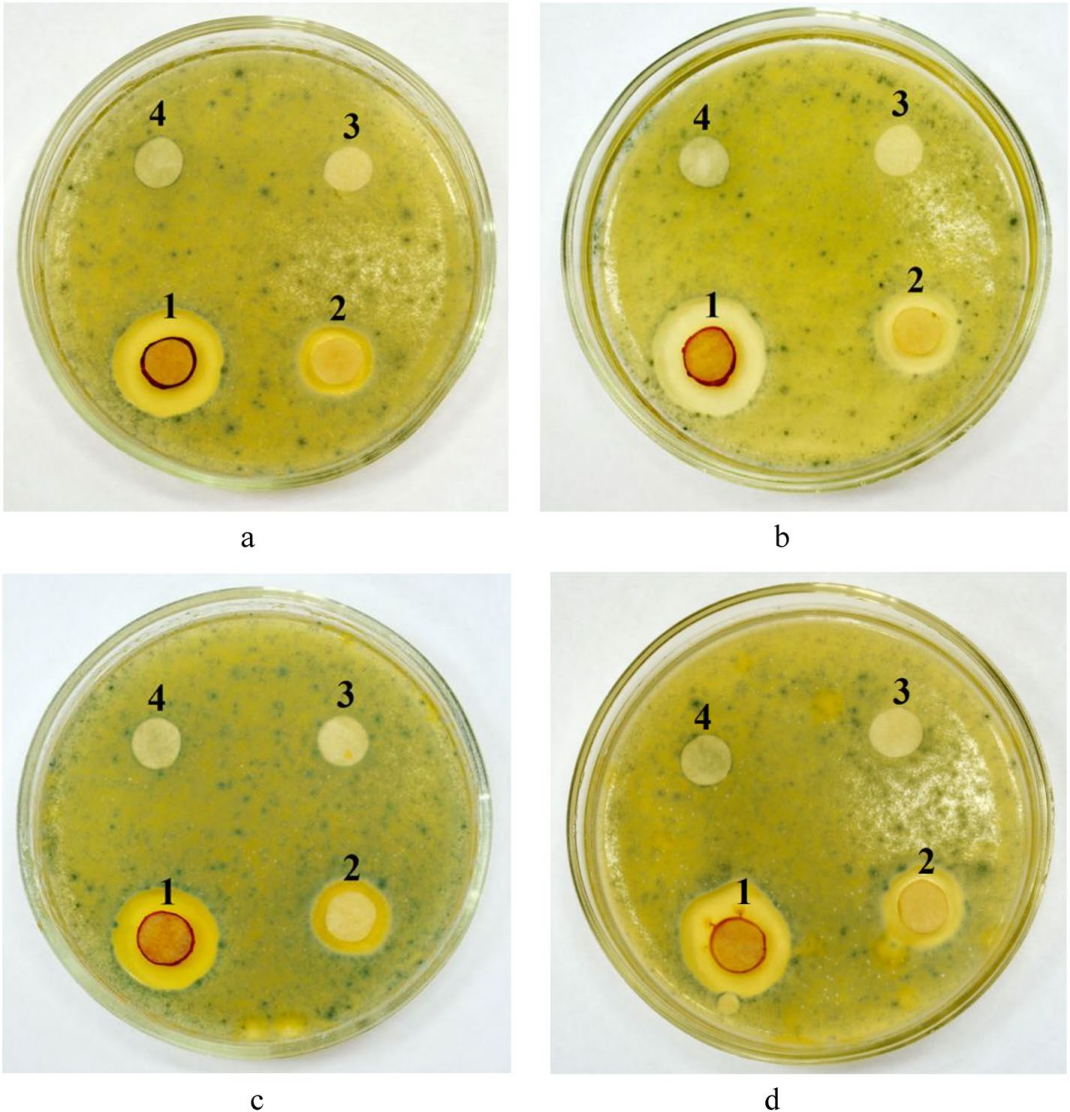
Investigation of the effect of concentration of disinfectant component—Ag NPs-DDAB (1—0.5 mg/mL, 2—0.05 mg/mL, 3—0.005 mg/mL, 4—0.0005 mg/mL) and type of detergent base (**a**) tap water, (**b**) cheese WM, (**c**) curd WM, (**d**) casein WM) on *Penicillium roqueforti*.

In addition to the effective cleaning of equipment from milk components, the detergent-disinfectant agent should be characterized by antimicrobial activity^60^. The study of the antimicrobial activity of prepared solutions in relation to *Penicillium roqueforti* showed that the nature of the solvent has a significant effect on the resulting activity (Fig. 9).

**Fig. 10:**
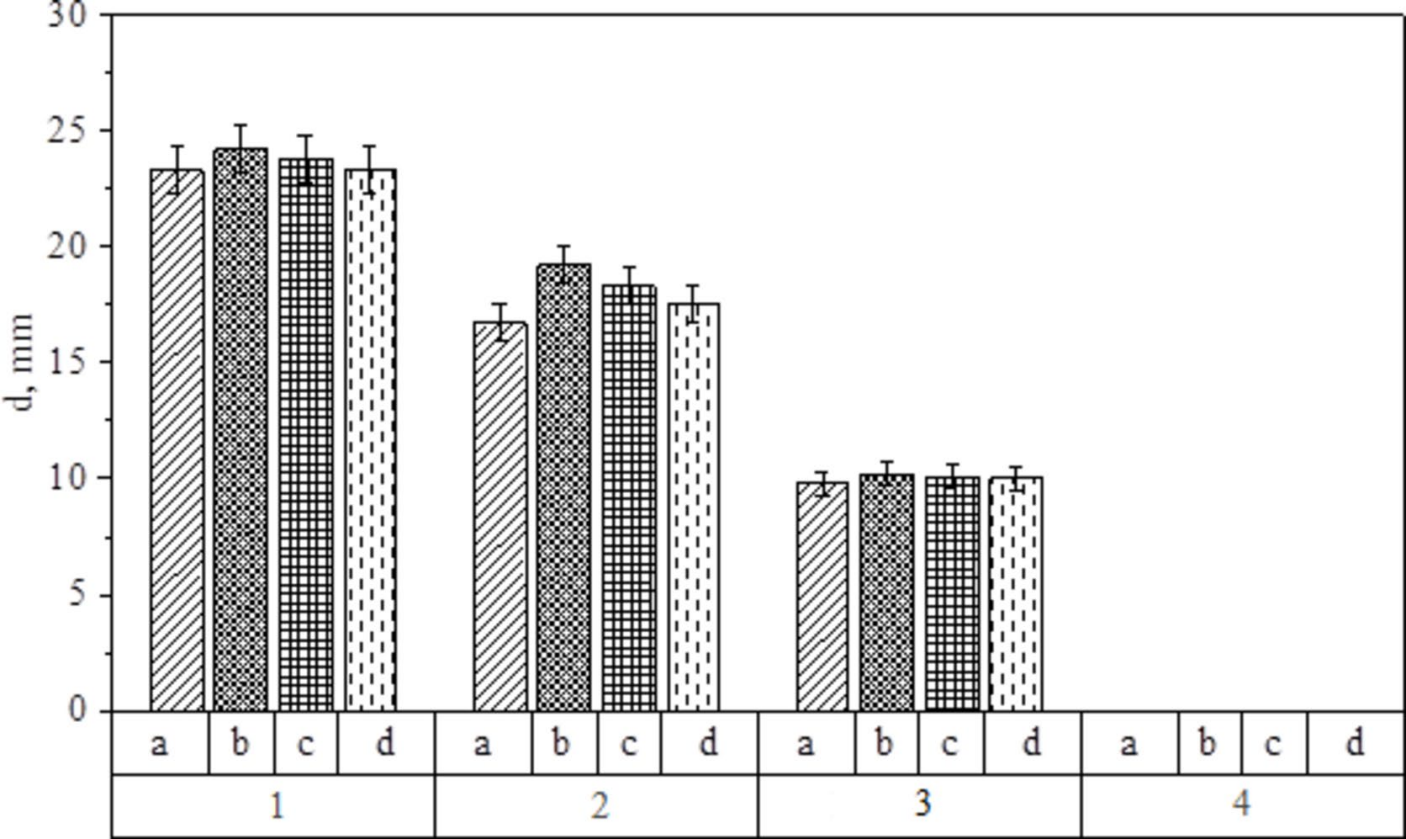
The dependence of the diameter of *Penicillium roqueforti* suppression zones on concentration of disinfectant component—Ag NPs-DDAB (1—0.5 mg/mL, 2—0.05 mg/mL, 3—0.005 mg/mL, 4—0.0005 mg/mL) and type of detergent base (a—tap water, b—cheese WM, c—curd WM, d—casein WM).

As shown in Fig. 9, the diffusion of a detergent-disinfectant agent into the nutrient medium results in the formation of a zone of suppression of *Penicillium roqueforti* growth around the disks. Thus, Ag NPs-DDAB has a suppression zone of 35 mm at concentration of 0.5 mg/mL and 25 mm at 0.05 mg/mL. Samples with concentration of 0.005 mg/mL and 0.0005 mg/mL did not show significant antibacterial activity. Notably, tap water as detergent base provided suppression zone of 35 mm, cheese WM–24 mm, and curd WM and casein WM did not inhibit *Penicillium roqueforti* growth. Figure 10 shows the dependence of the diameter of the suppression zones on the concentration of disinfectant component and the type of detergent base.

As can be seen in Fig. 10, samples with concentrations of Ag NPs-DDAB 0.5 mg/mL, 0.05 mg/mL and 0.005 mg/mL significantly inhibit the vital activity of *Penicillium roqueforti* regardless of the type of solvent (from 10 to 24 mm), but at concentrations of Ag NPs-DDAB 0.0005 mg/mL detergent-disinfectant agent does not exhibit antimicrobial activity, which corresponds to results of Panáček et al.^61^. It should be emphasized that tap water samples had the least influence. The remaining samples’ antimicrobial activity is listed in increasing order: casein WM, curd WM, and cheese WM. This finding correlates well with the ionic composition of solvents because ions contribute to the coagulation and aggregation of Ag NPs, lowering the detergent-disinfectant agent’s antimicrobial effectiveness.

The detergent-disinfectant agent based on cheese WM had the highest antibacterial activity due to the lowest concentration of different ions in its composition. The concentration of ions (Ca^2+^ and PO_4_^3−^) in curd WM is larger, as is their charge, and the coagulating action of Ag NPs is stronger than in cheese WM, according to the Schulze-Hardy rule^62^. Lower fungicidal activity was determined in casein WM with high concentration of Cl^–^ and Ca^2+^ and in tap water with high concentration of Ca^2+^, Mg^2+^, HCO_3_^–^^27^.

The acquired data allowed us to proceed to the next step of the experiment and evaluate the quality of hygienic treatment of the work surfaces. Table 4 shows the results of a comparative investigation of the efficacy of created samples and commercial detergent.

**Table 4:** Study of the quality of sanitary treatment of the work surfaces.

Sample	Work surface	Microbiological indicator		
		Coliforms	Total bacterial count	Pathogenic microorganisms, including salmonellas
Commercial detergent	Glass	Not detected	Not detected in 1 mL	Not detected
	Steel	Not detected	Not detected in 1 mL	Not detected
	Aluminum	Not detected	30 CFU/mL	Not detected
Curd WM with Ag NPs-DDAB	Glass	Not detected	Not detected in 1 mL	Not detected
	Steel	Not detected	Not detected in 1 mL	Not detected
	Aluminum	Not detected	10 CFU/mL	Not detected
Cheese WM with Ag NPs-DDAB	Glass	Not detected	Not detected in 1 mL	Not detected
	Steel	Not detected	Not detected in 1 mL	Not detected
	Aluminum	Not detected	10 CFU/mL	Not detected

Coliforms and harmful bacteria were not found on work surfaces, according to the data analysis. TBC concentrations of 30 CFU/mL (commercial detergent) and 10 CFU/mL (both experimental solutions) were found on the metal surface. Regarding the surfaces used in food production, this indication is not controlled. The findings are supported by research by the studies of Panáček et al.^61^, González-Fernández et al.^63^ and other scientists who examined the antibacterial activity of Ag NPs. The results obtained are consistent with those of the investigation of antimicrobial activity. As a result, it was discovered that WM had no effect on Ag NPs, and that the antibacterial activity of DDAB and the detergent-disinfectant agent may be compared to that of commercial detergent when used in everyday life.

The effectiveness of the sanitary treatment of work surfaces was evaluated before research of the corrosion activity of the detergent-disinfectant agent was conducted. Table 5 displays the study’s findings.

**Table 5:** Study of the corrosion activity of detergent-disinfectant agent.

Solvent of Ag NPs-DDAB	Stainless steel		Aluminum	
	Temperature, °C	Corrosion rate, mm/year	Temperature, °C	Corrosion rate, mm/year
Tap water	20	0.0041	20	0.0012
	35	0.0052	35	0.0021
	50	0.0074	50	0.0032
Distilled water	20	0.0035	20	0.0015
	35	0.0040	35	0.0018
	50	0.0066	50	0.0022
Curd WM	20	0.0051	20	0.0011
	35	0.0068	35	0.0020
	50	0.0075	50	0.0031
Cheese WM	20	0.0033	20	0.0013
	35	0.0054	35	0.0016
	50	0.0069	50	0.0028
Casein WM	20	0.0043	20	0.0011
	35	0.0067	35	0.0019
	50	0.0074	50	0.0032

*p* < 0.05 between all groups.

It has been determined that the corrosion activity of detergent-disinfectant agents is at the level of low-corrosive substances^64^ and does not correlate with the type of solvent, but instead increases with rise in washing process temperature, which should be taken into account during industrial implementation along with the potential accumulation of scum^65^.

Prior to making suggestions for industrial implementation, it is crucial to take into account the toxicity of the detergent-disinfectant agent. We investigated the toxicity of this on zebrafish, *Danio rerio*. Table 6 displays the research’s findings.

**Table 6:** Results of determination of acute toxicity of detergent-disinfectant agent and commercial alkaline detergent on *Danio rerio*.

Hydrobiont	Group	Dose of Ag NPs-DDAB mg/L	Number of fish in the group at the beginning of the experiment	Nnumber of dead fish	Number of surviving fish	Average number of surviving fish	Lethality, %	Probits
*Danio rerio*	*Commercial alkaline detergent*							
	1	100,000	5	5	0	0.0	100.0	7.32
	2	100,000	5	5	0			
	3	100,000	5	5	0			
	4	10,000	5	5	0	0.33	93.3	6.48
	5	10,000	5	4	1			
	6	10,000	5	5	0			
	7	1000	5	3	2	1.67	66.7	5.25
	8	1000	5	4	1			
	9	1000	5	3	2			
	10	100	5	0	5	4.33	13.3	3.88
	11	100	5	1	4			
	12	100	5	1	4			
	13	10	5	0	5	4.67	6.7	3.4
	14	10	5	1	4			
	15	10	5	0	5			
	*Detergent-disinfectant agent (cheese WM with Ag NPs-DDAB)*							
	1	100,000	5	5	0	0.0	100.0	7.32
	2	100,000	5	5	0			
	3	100,000	5	5	0			
	4	10,000	5	5	0	0.33	93.3	6.48
	5	10,000	5	5	0			
	6	10,000	5	4	1			
	7	1000	5	4	1	1.67	66.7	5.44
	8	1000	5	3	2			
	9	1000	5	3	2			
	10	100	5	1	4	3.67	26.7	4.38
	11	100	5	2	3			
	12	100	5	1	4			
	13	10	5	0	5	4.33	13.3	3.88
	14	10	5	1	4			
	15	10	5	1	4			

**Fig. 11:**
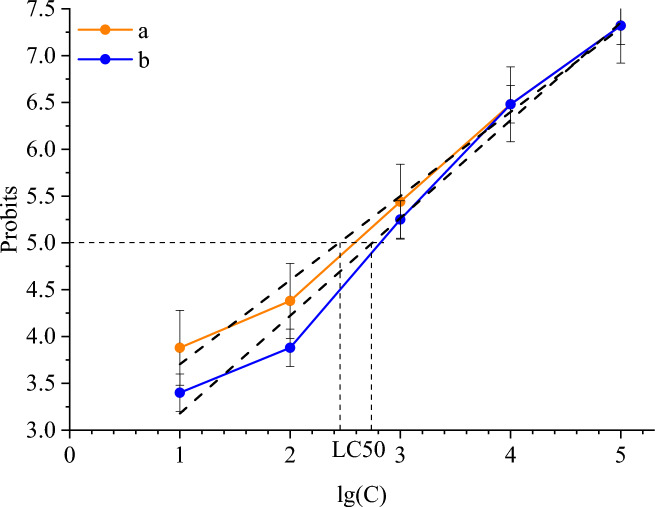
Probit Analysis of toxicity of detergent-disinfectant agent (**a**) and commercial alkaline detergent (**b**) on *Danio rerio*.

Figure 11 displays the outcomes of the probit analysis used to determine the average fatal concentration of a detergent-disinfectant agent and commercial alkaline detergent.

In line with Fig. 11, the average lethal concentration (LC50) for commercial alkaline detergent was 445 mg/L and for detergent-disinfectant agent it was 315 mg/L. As a result, albeit of the same order, the LD50 of the produced detergent-disinfectant agent is marginally lower than that of the reference detergent. The presence of Ag NPs can be used to explain the detergent-disinfectant agent’s increased toxicity. The recognized harmful impact is, however, less significant than in the works of Marinho et al.^66^ and Qiang et al.^67^, who studied the toxic effect of Ag NPs coated with polyvinylpyrrolidone and oleic acid on *Danio rerio*. It is significant to note that, as shown in Fig. 11, there were no changes in fish behavior when the samples were diluted by 10–100 times.

Because the concentration of the produced detergent-disinfectant agent in wastewater flushing will be considerably lower than the poisonous effect’s range, the detergent-disinfectant agent shouldn’t be hazardous to living things or the environment.

### Practical recommendations

**Fig. 12:**
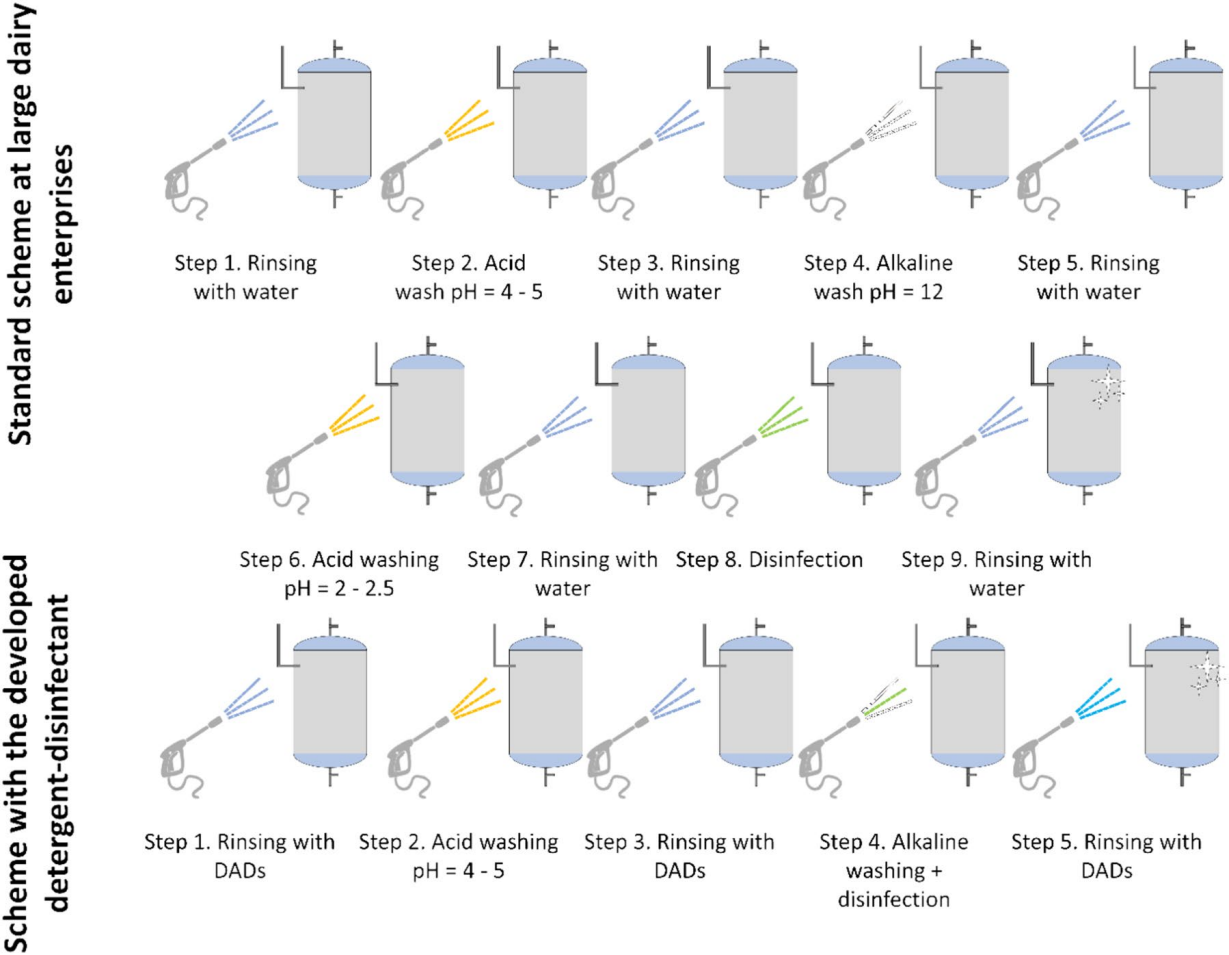
Block diagram of the process of sanitary treatment of work surface of technological equipment with detergent-disinfectant agent (DAD).

Based on the findings, we have presented a block diagram of the procedure for sanitizing the work surfaces of dairy industry technology (Fig. 12).

The experiment’s findings indicate that, while employing the created detergent-disinfectant agent, it is advised to entirely swap out the water used for washing, rinsing, and disinfection procedures with WM, which has proven to be the best solvent for Ag NPs-DDAB. At the same time, the amount of WM used for a single washing cycle will not be greater than 5 m^3^ (using the Stavropol Dairy Plant in Stavropol, Russia as an example).

An electrodialysis unit’s circuits can be cleaned using a detergent-disinfectant chemical. In this instance, it is advised to store the detergent-disinfectant agent in a container or to pump it through an adsorption column to absorb Ag NPs after washing. After the column, the detergent-disinfectant agent can be poured into the drain, and Ag NPs are then injected into a different container following column regeneration. To stop Ag NPs from entering the wastewater and having an even greater negative influence on the ecosystem, wastewater drains must have filter catchers.

As a result, using the developed detergent-disinfectant agent is economically justified without lowering the standard of sanitary treatment of the work surface of technological equipment, containers, and inventory. Additionally, there is a savings of enterprise resources, including water, electricity, time, and financial resources for the purchase of pricey detergents and disinfectants. An important aspect when using Ag NPs is compliance with the instructions for installing trap filters in sewers, since as a result, the load on wastewater will be reduced due to the rejection of surfactants, and this will have a positive effect on the environment.

## Conclusions

Using WM and Ag NPs, we developed a detergent-disinfectant agent for the first time in this study. For this, a procedure for creating Ag NPs stabilized with DDAB was developed and refined. It was discovered that a positively charged AgBr sol forms in the range of high AgNO_3_ concentrations (from 0.005 to 0.008 mol) and low DDAB concentrations (from 0.00001921 to 0.0004 mol), and that a negatively charged AgBr sol forms with an increase in Br^–^ concentration. Ag NPs stabilized with DDAB had a face-centered cubic crystal lattice with space group Fm-3m. In accordance with photon correlation spectroscopy, the particles’ average hydrodynamic radii were 4.5 and 20 nm, whereas their average diameters were 35 nm for acoustic spectroscopy and 40–50 nm for AFM and SEM, respectively. Although the investigation of the ζ-potential revealed that Ag NPs-DDAB were stable (ζ =  + 25 mV) throughout the pH range of 2–11, the analysis of the integral and differential titration curves of Ag NPs-DDAB indicated very alkaline reaction of the medium (pH ≈11).

A study of the acute toxicity of Ag NPs-DDAB in mice revealed that the LD50 = 4230 g/kg, LD84 = 4543 g/kg, LD100 = 4800 g/kg, and SLD50 =  ± 11.43. According to the degree of accumulation, Ag NPs-DDAB are classified as low-hazard compounds with moderate accumulation and no inhalation toxicity. The detergent-disinfectant agent that was created was highly antimicrobial active and had a rinsability of about 90%. Notably, the created detergent-disinfectant agent proved to be a low-corrosive chemical and displayed lower toxicity (315 mg/L) to *Danio rerio* than a commercial alkaline detergent (445 mg/L).

The experiment’s outcome was that the use of created detergent-disinfectant agents in dairy businesses would enable them to fully forgo the use of water for cleaning equipment, cut their washing expenses, and boost their production’s profitability. The proposed detergent-disinfectant agent can be used to clean an electrodialysis unit’s circuits, enabling the utilization of secondary waste from membrane milk processing and promoting resource efficiency and cleaner production in the dairy industry. In future perspective, it is planned to synthesis and study complexes of silver nanoparticles with other quaternary ammonium salts (alclidimethylbenzylammonium chloride, cetyltrimethylammonium chloride) for comparative analysis of their stability and detergent and disinfectant properties.

## Supplementary Information

Supplementary Information.

## Data Availability

The data used to support the findings of this study are included within the article.
