# Supplementary material for: Sustainable detergent-disinfectant agent based on whey mineralizate and silver nanoparticles for cleaner production in dairy industry
Source: Sci Rep. 2024 Oct 13;14:23943. doi: 10.1038/s41598-024-71542-9 (PMC11471832; doi:10.1038/s41598-024-71542-9)
Supplement: Supplementary file 1 — Supplementary Information. [file 41598_2024_71542_MOESM1_ESM.docx]

**Supplementary**

**S1. Optimization of the synthesis process of Ag NPs**

Table S1. Levels of variation of the main variable parameters

| Parameter | Parameter designation | Levels of variable variation | | | |
| --- | --- | --- | --- | --- | --- |
| m(AgNO_3_) : m(DDAB) | a | 0.3075 | 0.615 | 1.23 | 2.46 |
| ω (DDAB), % | b | 0.0076 | 0.0305 | 0.1219 | 0.4875 |
| the flow rate (υ), mL/min | c | 1.6 | 4 | 10 | 25 |
| n(NaВH_4_) : n(AgNO_3_) | d | 1 | 1.5 | 2 | 2.5 |

Table S2. Numerical values of variable parameters

| № | a | b | c | d |
| --- | --- | --- | --- | --- |
| 1 | 0.3075 | 0.0076 | 1.6 | 1 |
| 2 | 0.3075 | 0.0305 | 4 | 1.5 |
| 3 | 0.3075 | 0.1219 | 10 | 2 |
| 4 | 0.3075 | 0.4875 | 25 | 2.5 |
| 5 | 0.615 | 0.0076 | 4 | 2 |
| 6 | 0.615 | 0.0305 | 1.6 | 2.5 |
| 7 | 0.615 | 0.1219 | 25 | 1 |
| 8 | 0.615 | 0.4875 | 10 | 1.5 |
| 9 | 1.23 | 0.0076 | 10 | 2.5 |
| 10 | 1.23 | 0.0305 | 25 | 2 |
| 11 | 1.23 | 0.1219 | 1.6 | 1.5 |
| 12 | 1.23 | 0.4875 | 4 | 1 |
| 13 | 2.46 | 0.0076 | 25 | 1.5 |
| 14 | 2.46 | 0.0305 | 10 | 1 |
| 15 | 2.46 | 0.1219 | 4 | 2.5 |
| 16 | 2.46 | 0.4875 | 1.6 | 2 |

**S2. Scheme of the acute toxicity experiment in mice**

Table S3. The scheme of the experiment and the results of the study of Ag NPs–DDAB toxicity in white mice

| Type of administration | Group | Dose of Ag NPs-DDAB, µg/kg | Number of animals at the beginning of the experiment | Number of dead animals | Number of surviving animals | Lethality, % | Probits |
| --- | --- | --- | --- | --- | --- | --- | --- |
| *per os* | 2 | 3600 | 10 | 0 | 10 | 0 | 3.04 |
|  | 3 | 3900 | 10 | 2 | 8 | 20 | 4.16 |
|  | 4 | 4200 | 10 | 4 | 6 | 40 | 4.75 |
|  | 5 | 4500 | 10 | 8 | 2 | 80 | 5.84 |
|  | 6 | 4800 | 10 | 10 | 0 | 100 | 6.96 |

The calculation of the average lethal dose was carried out according to the following formula (S1):

LD_50_ = ((А+В) × (М-Н)) / 200, (S1)

Where: А and В – values of adjacent doses, µg/kg

М and Н – the frequency of deaths of adjacent doses, %

200 – constant coefficient

With oral administration of Ag NPs–DDAB LD_50_ = ((7500 × 20) + (8100 × 20) + (8700 × 40) +(9300 × 20))/ 200 = 846000 / 200 = 4230 µg/kg

The values of LD_16_ and LD_84_ were determined by linear interpolation based on doses in mg and corresponding probits. Probit 4 corresponds to LD_16_ = 3857, and probit 6 corresponds to LD_84_ = 4543.

The error rate SLD_50_ was calculated using the formula (S2):

SLD_50_ = (LD_84_ – LD_16_) / 2n, (S2)

Where LD_16_ and LD_84_ – effect doses, µg/kg;

n – the total number of animals in groups for which the values of probits are in the range of 3.5-6.5;

SLD when calculating the acute toxicity of oral administration: (4543 - 3857) / (30 × 2) = 686 / 60 = 11.43.

**S3. Dynamics of changes in the mass of mice in an experiment for inhalation toxicity**

Table S4. Weight of mice after 14 days of observation, grams

| Animal | Control group | | Experimental group | |
| --- | --- | --- | --- | --- |
|  | before | after | before | after |
| 1 | 19.25 | 18.66 | 20.14 | 19.79 |
| 2 | 20.58 | 21.17 | 18.52 | 18.46 |
| 3 | 18.35 | 18.24 | 19.71 | 20.13 |
| 4 | 16.95 | 17.78 | 20.07 | 19.82 |
| 5 | 21.11 | 20.65 | 16.89 | 17.27 |
| 6 | 16.79 | 17.02 | 17.93 | 18.15 |
| 7 | 19.22 | 18.86 | 18.01 | 17.83 |
| 8 | 20.09 | 19.73 | 21.16 | 20.99 |
| 9 | 18.92 | 19.30 | 19.88 | 20.32 |
| 10 | 17.74 | 18.17 | 20.11 | 19.77 |

**S4. Investigation of the average hydrodynamic radius of Ag NPs–DDAB in various solvents**

To assess the possibility of application of Ag NPs–DDAB in accordance with the purpose of the work, study of the average hydrodynamic radius of Ag NPs–DDAB in various media was carried out. Distilled water, tap water, 1M and 5M NaCl solution, whey permeate and WM were used as solvents. The data obtained are shown in Figure S1.

Figure S1. The initial hydrodynamic radii formed by dissolving the concentrate of Ag NPs–DDAB in: 1) distilled water, 2) 1 M NaCl solution, 3) whey permeate, 4) cheese WM, 5) curd WM, 6) tap water, 7) 5 M NaCl solution

The analysis of the obtained data showed that Ag NPs–DDAB, which were dissolved in tap water (400 nm) and in 5 M NaCl solution (870 nm), have the largest average hydrodynamic radius.
